# Personalized assessment of high-definition slow-oscillatory transcranial direct current stimulation (so-tDCS) in older adults with sleep and subjective cognitive complaints: A study protocol for a randomized clinical trial

**DOI:** 10.1371/journal.pone.0360573

**Published:** 2026-10-13

**Authors:** Hanna Lu, Xi Ni, Natalie Shu Yang, Yuk Shan Yuen, Yuqi Gong, Suk Ling Ma

**Affiliations:** 1 Department of Psychiatry, The Chinese University of Hong Kong, Hong Kong SAR, China; 2 Department of Psychology, University of Greifswald, Greifswald, Germany; 3 Department of Linguistics, The Chinese University of Hong Kong, Hong Kong SAR, China; 4 Department of Neuroscience, School of Translational Medicine, Monash University, Melbourne, Australia; 5 School of Art and Humanities, Tung Wah College, Hong Kong SAR, China; Hangzhou Normal University, CHINA

## Abstract

**Background:**

Subjective cognitive decline (SCD) is increasingly recognized as an early and potentially modifiable stage in the trajectory of neurodegenerative diseases. In older adults with SCD, untreated sleep disturbances may accelerate the neurodegenerative process, potentially via mechanisms involving amyloid-beta accumulation, and contribute to more rapid cognitive deterioration. Currently, non-pharmacological treatment options that concurrently manage SCD and sleep disturbances remain scarce. High-definition slow-oscillatory transcranial direct current stimulation (so-tDCS) is an emerging, frequency-specific neuromodulation technique designed to enhance brain health and cognition.

**Methods and design:**

This is a randomized, double-blind, sham-controlled clinical trial. Right-handed Chinese older adults with SCD and sleep disturbances will be randomly assigned to receive either high-definition 0.75 Hz so-tDCS or sham stimulation over a 4-week intervention period, with 40 participants per group. Outcome measures, including galvanic skin response, subjective cognitive complaints, sleep quality, salivary amyloid-beta levels, and domain-specific cognitive performance, will be evaluated at four time points: baseline, week 4 (immediately post‑intervention), and follow‑up at weeks 8 and 12.

**Conclusions:**

Personalized neurophysiological signals, namely electrode-skin impedance, will be monitored using state-of-the-art measurement during so-tDCS. The successful implement of this signal will provide researchers with access to an “add-on” setting that enables them to easily, quickly and quantitatively evaluate real-time neurophysiological signals, thereby facilitating the incorporation of transcranial brain stimulation into clinical practice.

**Trial registration:**

ClinicalTrials.gov Identifier: NCT07433738

## Introduction

As the world undergoes rapid population aging, issues surrounding brain health and cognitive longevity have become a core concern in clinical consultations and rehabilitation practices [1]. Over the past decade, research focus has shifted from objective cognitive dysfunction to subjective cognitive decline (SCD), increasingly recognized as the earliest symptomatic stage of age-related neurodegenerative diseases [2]. With SCD affecting a considerable proportion of older adults (prevalence typically 20–50%) [3], evidence from longitudinal studies suggests that up to ~60% of individuals with SCD may progress to mild cognitive impairment (MCI) or dementia over a 15-year period [4,5]. Untreated SCD is frequently comorbid with sleep disturbances or major depression in the ageing population [6,7]. Notably, the prevalence of sleep disturbances among older adults with SCD has been reported to be as high as 64% [8]. Two additional cohort studies found that sleep disturbances were associated with severe subjective cognitive complaints [9,10]. Moreover, sleep disturbances can markedly impair the glymphatic function system and reduce clearance of amyloid-beta (A*β*), thereby increasing the risk of Alzheimer’s disease (AD) [11]. Accordingly, the coexistence of SCD and sleep disturbances in older adults may accelerate the progression of neurodegeneration, potentially leading to an earlier transition to MCI or dementia [12,13]. In light of SCD’s status as an early risk stage for neurodegeneration, effective management of SCD and its comorbidities assumes greater clinical importance, given their established association with an increased risk of accelerated cognitive decline.

While early intervention in SCD remains essential and clinically valuable, the substantial heterogeneity of SCD and its comorbidities poses significant therapeutic challenges and hinders effective management. The heterogeneity of SCD can be decomposed into two main levels: (1) Inter-individual level: SCD refers to a diagnostically heterogeneous population, with individuals varying widely in their patterns of subjective cognitive complaints (domain-specific vs. global) [1], types of comorbidities, and degree of neuropathological load (e.g., A*β* deposition) [14,15]. (2) Intra-individual level: This concerns personalized indicators of treatment outcomes. Intra-individual variability (IIV), reflecting the short-term fluctuation or variation in brain signals and cognitive performance, has recently been applied in published clinical trials as a sensitive measure and predictor of treatment response [16,17]. In the context of heterogeneity, growing evidence shows that the disturbed cortical slow oscillations can interfere with domain-specific cognitive function, A*β* synthesis and glymphatic clearance systems [18,19], leading to cognitive dysfunction and A*β* accumulation in aged mice [20], and human adults with SCD [21], sleep disorders [22] and dementia [23]. Particularly, recent findings highlight that those slow oscillations (<1 Hz) can facilitate neuronal synchronization in the process of enhancing cognition and preserving neuroplasticity [24]. This bidirectional relationship among slow oscillations, sleep and cognitive functions sheds new light on non-pharmacological intervention aimed at alleviating subjective cognitive complaints and sleep disturbances through modulating brain activities.

At present, non-pharmacological interventions are increasingly recommended as first-line treatments for early-stage cognitive decline [25]. However, clinical trials examining non-pharmacological interventions in older adults with SCD and related comorbidities remain scarce, and current evidence is insufficient to test its safety and efficacy at individual level [26]. As a non-invasive and cost-effective modality of neuromodulation, frequency-specific transcranial current stimulation is gaining increasing popularity due to its benefits on sleep quality and cognitive functions in older adults [27]. Of note, a neurophysiological indicator of frequency-specific transcranial current stimulation, namely impedance, refers to the skin resistance and reactance at the electrode-skin interface. The direct stimulator must overcome the electrode-skin impedance to deliver the current to the targeted brain regions. Impedance in transcranial current stimulation is of great importance to safety (e.g., skin redness), dose estimation, and efficacy. Thus, monitoring skin impedance is critical for both safety and the interpretation of treatment effects. Galvanic skin response (GSR) is a physiological measure of changes in the electrical properties of the skin, particularly skin conductance (the inverse of skin resistance) [28]. In the studies of slow-oscillatory transcranial current stimulation, GSR-related measures may partially reflect the skin-impedance component of the overall circuit and serve as a real-time safety monitoring system at the individual level.

Collectively, frequency-specific brain oscillations can be modulated by weak electrical current, shedding new light on the therapeutic advantages for older adults with co-occurring SCD and sleep disturbances. High-definition so-tDCS delivers a focal monophasic sinusoidal waveform at a low frequency that can proficiently entrain endogenous brain oscillations and modulate brain activities. Given the very limited therapeutic options for older adults suffering from SCD and sleep disturbances, we aim to conduct a randomized, double-blind, sham-controlled clinical trial to investigate the efficacy and sustainability of high-definition so-tDCS in treating SCD and sleep disturbances, as well as the clinical values of GSR-related measures in predicting individual’s treatment outcomes.

## Materials and methods

### Study design

This study adopts the design of a randomized, double-blind, sham-controlled clinical trial of high-definition so-tDCS. Assessments will be conducted at baseline, 4^th^ week, 8^th^ week, and 12^th^ week to evaluate the safety, efficacy and sustainability of high-definition so-tDCS effects on subjective cognitive complaints, sleep quality, salivary A*β* levels and cognitive functions (Fig 1). Primary outcomes will be the changes in skin impedance, subjective cognitive complaints and sleep quality features following so-tDCS intervention. Secondary outcomes will include changes in cognitive functions and salivary A*β* levels, as well as the relationships among the changes in impedance, subjective cognitive complaints, sleep quality and salivary A*β* levels. The study will be conducted following the Consolidated Standards of Reporting Trials (CONSORT) statement (http://www.consort-statement.org) (Supplement 1).

**Fig 1:**
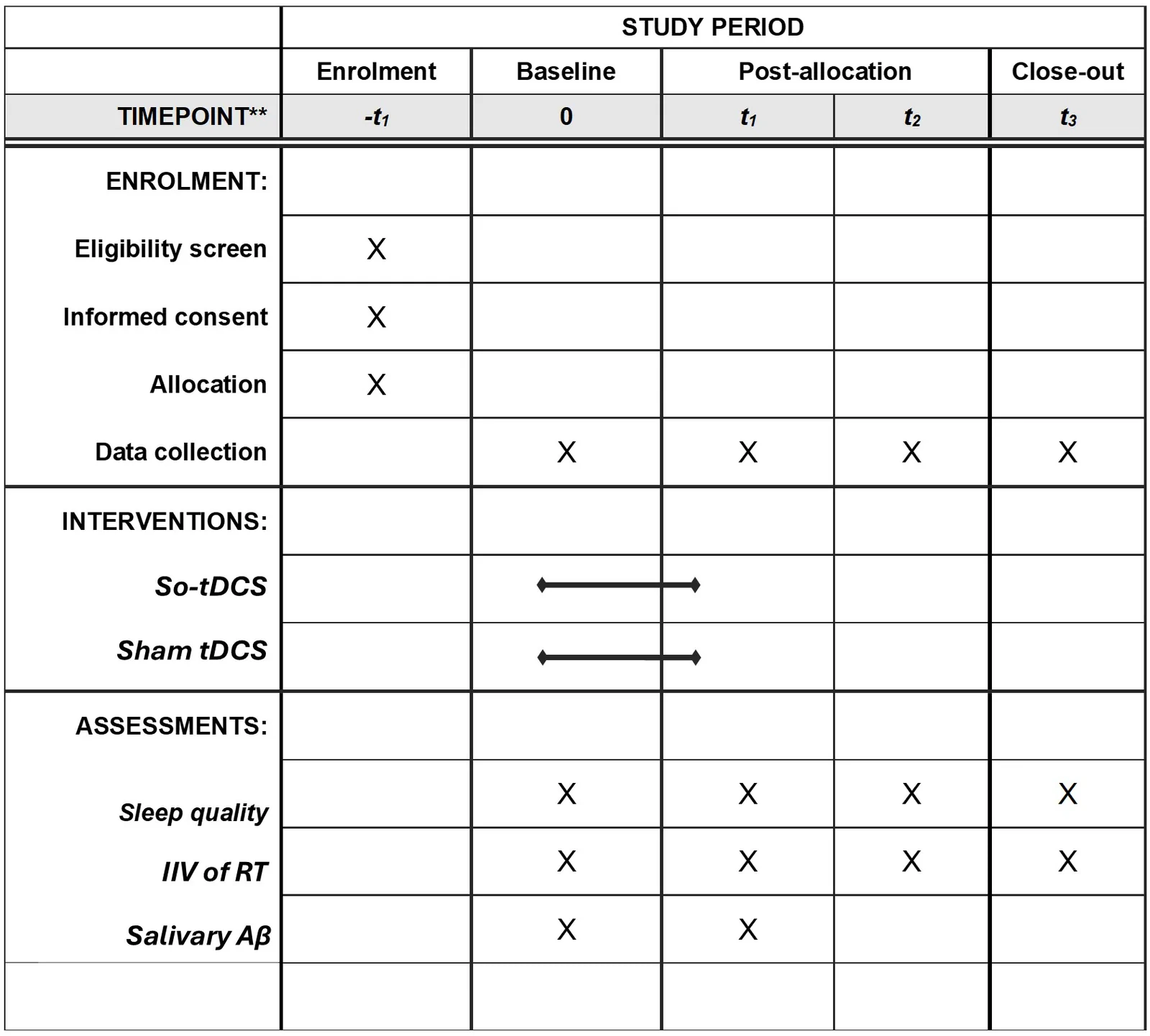
Schedule of the randomized clinical trial according to the standard protocol items: Recommendations for interventional trials checklist (SPIRIT). Abbreviations: so-tDCS = Slow-oscillatory transcranial direct current stimulation; A*β* = Amyloid-beta; IIV of RT = Intra-individual variability of reaction time.

### Study populations

Eligible participants will be recruited through a local trial-ready cohort (TRC), i.e., The Hong Kong cohort of abnormal sleep in ageing population (HK-ASAP) [29]. A multidisciplinary research team, including neuroscientist, psychologist, geriatrician, will identify the candidates who have subjective complaints of sleep and cognition, and will assess their eligibility and availability for participation in this clinical trial. Before participating in this study, both the participants and their families or caregivers will receive a comprehensive briefing about the study and will be required to sign the written informed consent. The research proposal can be found in Supplement 2

Eligible participants should meet the inclusion criteria as follows:

1. Chinese, right-handed, aged from 60 to 80 years.
2. Normal global cognition defined as a total score of the Montreal Cognitive Assessment Hong Kong version (HK MoCA) > 26 [17].
3. Subjective cognitive decline (SCD) is evaluated by a 10-item questionnaire that covers subjective cognitive complaints (SCC) in the core cognitive domains [30]. Participants will answer yes/no questions referring to the presence of cognitive deficits in the last six months. The answers of SCC are codified as 0 (absence of complaints) or 1 (presence of complaints). Answers will be summed up, and the total of complaints will be obtained ranging from 0 to 10. In this study, the status of SCD is defined as SCC total score > 1.
4. Sleep disturbances or poor sleep quality is assessed and defined with a total score of the Pittsburgh Sleep Quality Index (PSQI) greater than 5 [31].
5. No impairments in the activities of daily living.

Exclusion criteria include:

1. Previous diagnosis of age-related neurodegenerative diseases, such as Alzheimer’s disease and Parkinson disease.
2. History of neurological diseases including brain tumor, and stroke.
3. History of psychiatric disorders including schizophrenia and depression.
4. Presence of positive neurological diseases, such as stroke, brain tumor, dementia.
5. Physically unable to attend the sessions of intervention.
6. Currently enrolled in another intervention study.
7. Currently taking a psychiatric or other medication that may affect sleep quality and cognition.

### Ethical consideration

Strict ethical standards will be upheld throughout the clinical trial, including the provision of informed consent and the protection of participants’ anonymity, privacy, and confidentiality. Participants will be eligible for recruitment only if they are considered mentally fit to sign informed consent. No personally identifiable information, including name, dates of birth, or mobile phone numbers, will be disclosed in any reports or publications. Participants may withdraw from this study at any time, and such withdrawal will not affect their access to future services or care. Ethics approval has been obtained from the Joint Chinese University of Hong Kong New Territories East Cluster Clinical Research Ethics Committee (Joint CUHK NTEC-CREC) (Ref. No.: 2025.118). The protocol has been registered with the CCT Clinical Trials Registry of the Chinese University of Hong Kong and linked to ClinicalTrials.gov (Identifier: NCT07433738). The trial will be reported in accordance with the requirements of major international academic journals. The study will be conducted in compliance with the Declaration of Helsinki and the Good Clinical Practice guidelines of the International Conference on Harmonisation of Technical Requirements for Registration of Pharmaceuticals for Human Use (ICH-GCP).

### Pre-intervention assessments

#### Magnetic resonance imaging.

High-resolution structural MRI (MRI) scans will be collected at the Prince of Wales Hospital using a 3.0 Tesla Siemens MAGNETOM Prisma MRI scanner for detecting the major neurological diseases, such as stroke, brain tumor. T1-weighted magnetization prepared rapid gradient echo (MPRAGE) sequence is applied to optimize the grey-white contrast. The MPRAGE sequence yields high-quality isotropic images with the voxel size of 1 mm × 1 mm × 1 mm. The detailed parameters of this sequence are axial acquisition with a 256 × 256 × 192 matrix, field of view (FOV)=230 mm, repetition time (TR)=2070 ms, echo time (TE)=3.93 ms, thickness = 1 mm, no gap, flip angle = 15^◦^. All MRI scans will be imported to BrainSuite for quantitative analysis [32]. Surface-based morphometric measures, including cortical volume, thickness and folding, are employed as brain features to precisely localize the stimulation target, correlate with neurophysiological signals and control the baseline brain features in two randomized groups.

#### Randomization and masking.

As shown in Fig 2, eligible participants will be randomly assigned in a 1:1 ratio to one of two intervention groups: 1) High-definition so-tDCS; 2) Sham tDCS. To ensure balanced allocation across treatment groups, a randomization assignment will be generated prior to enrollment using an online system by a statistician who is not involved in the study design. Both outcome assessors and participants will be blinded to group allocation.

**Fig 2:**
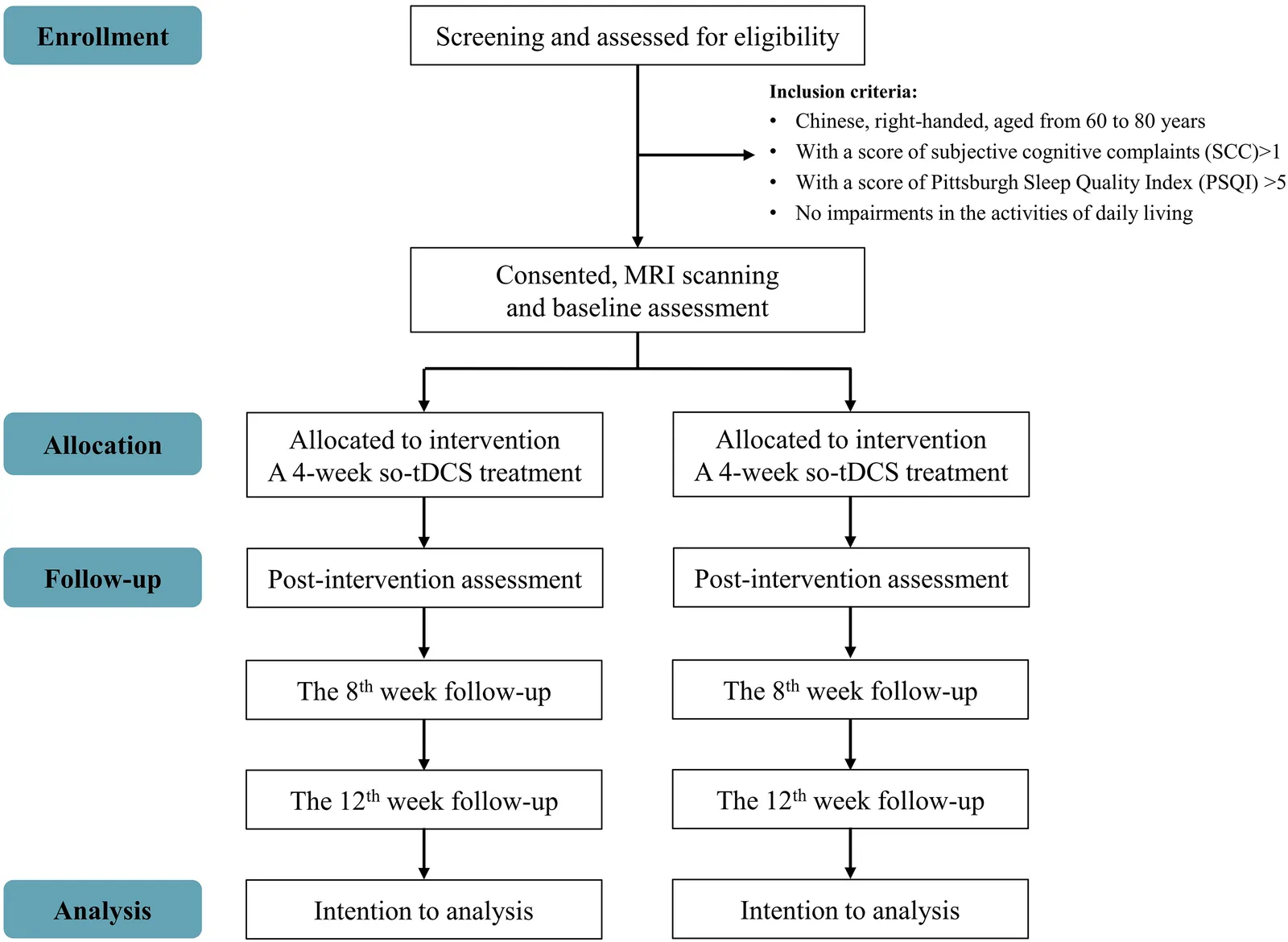
The Consolidated Standards of Reporting Trials (CONSORT) flow diagram for the clinical trial of slow-oscillatory transcranial direct current stimulation. Abbreviations: so-tDCS = Slow-oscillatory transcranial direct current stimulation; MRI = Magnetic resonance imaging.

### Intervention strategies

#### Apparatus and treatment setting.

The so-tDCS is delivered by a battery driven direct current stimulator (DC-Stimulator Plus, NeuroConn, Ilmenau, Germany). The high-definition setting is applied by placing an anodal electrode over the center of the treatment target and placing four cathodal electrodes surrounding the anodal electrode. Each electrode in the “4 × 1” montages had a diameter of 1.2 cm. We place the anodal electrode over the center of left dorsolateral prefrontal cortex (DLPFC) (i.e., F3 according to the international 10–20 EEG system) as used in previous studies that investigated preserved cognition [33] and placed the four return electrodes around anodal electrode. For safety and health considerations, single use sponge electrodes (1.2 cm) will be applied in this study. Participants will be instructed to relax during the intervention of high-definition so-tDCS.

#### Stimulation modalities.

High-definition so-tDCS: 20 minutes at 0.75 Hz 2 milliamps [33].

Sham high-definition so-tDCS: With a same setting of high-definition as active so-tDCS, the stimulation of sham so-tDCS only lasts for 30 seconds. The electrodes will be left in place for a further 20 minutes. The purpose of this sham procedure is to mimic transient skin sensation of tingling induced by current stimulation, but without producing any sustainable effects.

### Intervention schedule

The duration of the intervention is four weeks, with three sessions per week, 20 minutes per session. All participants will receive a total of 12 sessions of high-definition so-tDCS.

### Outcome assessments

#### Primary outcomes.

1. Skin impedance in so-tDCS: Neurophysiological signal recorded and measured by a built-in wearable Galvanic Skin Response (GSR) sensor. GSR sensor is placed on the fingers to detect the impedance of the skin when the participant is receiving so-tDCS intervention.
2. Subjective cognitive complaints (SCC): a 10-item questionnaire that covers subjective complaints in the core cognitive domains [30]. Participants will answer ten yes/no questions relating to the presence of cognitive complaints in the last six months. The answers of SCC are codified as 0 (no complaints) or 1 (presence of complaints). Answers will be summed up, and the total of complaints will be obtained ranging from 0 to 10.
3. Subjective sleep quality: The 19-item self-reported questionnaire of the Pittsburgh Sleep Quality Index (PSQI) is used to assess subjective sleep quality [31]. The 19 items produce seven component scores. The total score of these components yields a measure of subjective sleep quality that can be used to determine the status of sleep disturbances. The cutoff score of sleep disturbances is 5 or more [34].

#### Secondary outcomes.

1. Actigraphic records were used to quantify sleep-wake cycle and estimate the objective sleep efficiency [35,36]. The actigraphy device (Philips Actiwatch 2) is about the size of a wristwatch and is usually worn on the wrist continuously for multiple days and nights, which can be used as an objective measurement of sleep quality and sleep-wake cycle in older adults.
2. Intra-individual variability of reaction time (IIV-RT), representing the within-person fluctuations, is calculated based on the performance of attention network test (ANT). For correcting the increased RT in elderly, Intraindividual coefficient of variation of reaction time (ICV-RT) is used for assessing the IIV-RT enumerated with the formula: ICV-RT = (Standard deviation of RT/mean RT) × 100 [17].
3. The levels of salivary A*β*: At the baseline and 4^th^ week, salivary samples will be collected at a consistent time of day to avoid circadian effects and will be kept on ice during the collection. The levels of A*β*42 and A*β*40 in the plasma samples will be quantified by enzyme-linked immunosorbent assay (ELISA)-type assays (Aurin Biotech, Inc.) [37]. Each sample will be analyzed in duplicate.

### Assessment schedule

Comprehensive assessments, including SCC, sleep quality, IIV of RT and adverse events, will be conducted at one week before intervention (baseline, T0), 4^th^ week (T1), 8^th^ week (T2), and 12^th^ week (T3). Salivary A*β*42 and A*β*40 will be assessed at baseline and 4^th^ week.

### Sample size estimation

The sample size is estimated using G*Power (version 3.1.9.7). In line with the primary aims of this study, the estimation is based on comparisons of the PSQI total score between two randomized groups and across time points. The anticipated effect size for improvements in subjective sleep quality resulting from repeated active tDCS is derived from our previous findings in a tDCS intervention study conducted in patients with mild neurocognitive disorder due to Alzheimer’s disease [33]. A 4-week course tDCS showed significant positive effects on subjective sleep quality, as reflected by a mean change of 2.36 ± 1.51 in the PSQI total score [38]. Based on the proposed statistical analysis examining the fixed effects of so-tDCS on the PSQI total score, we set the desired power (1-*β*) as 0.8 and the confidence level as 0.95. Thirty-six participants per intervention group will be needed to achieve a statistical power of 0.8. Accounting for an anticipated dropout rate of approximately 13%, as reported in previous clinical trial [33], we plan to recruit 40 participants per group, resulting in a total sample size of 80 participants for this clinical trial.

### Statistical analyses

Analyses will be on an intention-to-treat basis. Linear mixed models will be applied to assess and quantify the differences between the pre- and post-intervention time points on the primary and secondary outcome measures. This statistical method will facilitate the inclusion of participants with missing data. Time (i.e., follow-up period) will be modelled as fixed effects. Participants will be modelled as random effects at different time points. Score changes of subjective cognitive complaints, neurophysiological features, sleep quality, cognition and salivary A*β* levels from baseline to follow-up time points will be tested with occasions (i.e., time points) at level one and participants at level two. For better controlling the practice effects, generalized estimating equations will be used in the analysis of repeated measurements of treatment outcomes. Covariates identified from baseline differences will be entered in the regression model. Secondary analyses of the predictive relationships between cognitive complaints, sleep quality, *β*-amyloid levels and neurophysiological features will be performed. Multiple comparisons across the three primary outcomes will be corrected using false discovery rate (FDR) approach. Statistical significance will be set at 2-sided *p* < 0.05. Computations will be performed using R Studio (version 1.1.456).

### Study timeline

The clinical trial is scheduled to take place from the 01/10/2026–01/10/2028. We anticipate that participant recruitment will be started from 01/10/2026 and completed by 01/03/2027. Data collection and interventions, including MRI scanning and assessments of sleep quality and cognitive functions, are expected to conclude by 01/01/2028. Following data cleaning and analysis, we anticipate disseminating the main study results by 01/10/2028.

### Data management and quality control

Each enrolled participant will be assigned a unique, sequential study identification number. The questionnaires, laboratory samples, and data files will be identified solely by this unique ID to ensure de-identification and protect participant confidentiality. All study-related data will be stored on secure, encrypted hard disks with restricted, role-based access. Regular system backups will be performed to prevent data loss. All personnel, including investigators, research assistants, and data entry staff, will receive comprehensive, documented training on the study protocol, the manual of so-tDCS operations, and specific training on the procedures of data entry. Only trained and authorized personnel will have access to the system.

## Discussion

This paper presents the rationale and study protocol for a randomized, double-blind, sham-controlled clinical trial. Slow-oscillatory tDCS (so-tDCS) is a targeted non-invasive method designed to exogenously boost slow oscillations, primarily to enhance sleep physiology and sleep-mediated cognitive process like memory consolidation. We hypothesize that individuals receiving so-tDCS will demonstrate greater improvements in subjective cognitive complaints and sleep quality. We anticipate that these improvements will be associated with reduced IIV of RT and salivary levels of A*β*40 and A*β*42. Moreover, the mean impedance recorded during so-tDCS intervention will be used as a digital marker for monitoring safety and a predictor for therapeutic response. This study offers a novel, neuroscience-driven approach to the non-pharmacological and non-invasive management of common comorbidities in older adults.

### Strengths and limitations

This double-blind, sham-controlled clinical trial will add a valuable amount of data regarding cognitive and sleep complaints, skin impedance, and salivary A*β* levels in older adults, and will address some of the prior weakness in this field. Importantly, this study may provide an in-depth understanding of skin impedance during so-tDCS and its physiological significance, offering in vivo research data for scientists and clinicians.

While saliva-based A*β* testing represents a non-invasive and cost-effective method of investigating early-stage Alzheimer’s disease in research settings, it is hindered by notable limitations that may compromise its reliability. The measurements of A*β* in saliva samples display considerably higher analytical variability compared to those obtained from plasma or cerebrospinal fluid (CSF). This increased variability stems primarily from the much lower concentrations of the biomarker in saliva samples, which challenge detection sensitivity and precision, as well as from greater vulnerability to oral contamination (e.g., from food residues, bacteria, or gingival crevicular fluid) that can introduce artifacts and confound results. These factors may contribute to inconsistencies in results and reduced reliability compared to more established biofluids. Although actigraphy is used in this study, the lack of polysomnography (PSG) constrains our ability to explore the mechanistic pathways linking slow-wave sleep, glymphatic function and cognition, especially given the well-established association between poor sleep quality and impaired glymphatic clearance [39–41]. Additionally, because genetic data were not available in this clinical trial, our analyses could not adjust genetic risk factors, particularly APOE genotype that may influence the effects of so-tDCS.

## Supporting information

S1 File SPIRIT checklist. (PDF)

S2 File Research protocol. (PDF)
