# Supplementary material for: Personalized assessment of high-definition slow-oscillatory transcranial direct current stimulation (so-tDCS) in older adults with sleep and subjective cognitive complaints: A study protocol for a randomized clinical trial
Source: PLoS One. 2026 Oct 13;21(10):e0360573. doi: 10.1371/journal.pone.0360573 (PMC13660898; doi:10.1371/journal.pone.0360573)
Supplement: Supplementary file 2 [file pone.0360573.s002.pdf]

Research Protocol

Personalized assessment of high-definition slow-oscillatory transcranial direct current stimulation (so-tDCS) in older adults with subjective cognitive and sleep complaints

(i) Research context

As the world is ageing rapidly, concerns about brain health and longevity are becoming a central topic that arises during medical consultations and rehabilitations [1]. Indeed, the past decade has witnessed the shifted research interests from objective cognitive deficits to subjective cognitive decline (SCD), which could reflect the recognition of SCD as the earliest symptomatic manifestation of age-related neurodegenerative diseases [2]. With a relatively high prevalence (i.e., 25%-50%) of SCD in older adults [3], about 60% of them may convert to mild cognitive impairment (MCI) or dementia over a 15-year period [4,5]. Untreated SCD is often comorbid with sleep disturbances or depression in late adulthood [6,7]. Of note, the prevalence of sleep disturbances in elderly with SCD has been reported as high as 64% [8]. Another two cohort studies found that sleep disturbances, as a risk factor, were related to the severity of subjective cognitive complaints [9,10]. Moreover, sleep disturbances can severely influence the clearance of amyloid beta (Aβ) and tau, and further increase the risk of Alzheimer's disease [11]. Thus, the co-occurrence of SCD and sleep disturbances in older adults may accelerate the progression of neurodegeneration and lead to the early onset of MCI or dementia [12,13]. In light of the early risk stage, the management of SCD and its comorbidity becomes more important for clinicians because of its associations with an increased risk of future accelerated cognitive decline.

While early intervention for SCD is certainly critical and clinically meaningful, the therapeutic challenges associated with heterogeneity hamper the successful management of SCD and its comorbidity. The heterogeneity in SCD can be decomposed into two levels: (1) Inter-individual level: Components of subjective cognitive complaints. The diagnosis of SCD refers to a heterogeneous population with comorbidity, domain-specific complaints [1] and different neuropathological loads (i.e., Aβ and tau levels) [14,15]. (2) Intra-individual level: Individualized indicators of treatment outcomes. Intra-individual variability (IIV), representing the short-term fluctuations of brain signals and cognitive performance, is used to measure and predict the treatment responses in recent published clinical trials [16,17]. In the context of heterogeneity, growing evidence shows that the disturbed cortical slow oscillations can interfere with domain-specific cognitive systems, β-amyloid synthesis and clearance systems [18,19], leading to cognitive dysfunction and β-amyloid accumulation in aged mice [20], SCD [21], sleep disorders [22] and dementia [23]. More importantly, recent findings highlight that slow oscillations (i.e., <1 Hz) could facilitate neuronal synchronization in the process of enhancing cognitive functions and preserving neuroplasticity [24]. This bidirectional relationship between slow oscillations, sleep and cognitive function sheds new light on the non-pharmacological intervention to alleviate subjective cognitive complaints and sleep disturbances through modulating the brain activities.

At present, non-pharmacological interventions are increasingly recommended as the first-line treatments for early-stage cognitive impairments [25]. However, clinical trials examining the non-pharmacological interventions in older adults with SCD and related comorbidities are very few, and current clinical evidence is lacking for testing its efficacy and sustainability at individual level [26]. As a non-invasive and cost-effective form of non-invasive brain stimulation, frequency-specific transcranial current stimulation shows increasing popularity because of its benefits on sleep quality and cognitive functions in older adults [27].

Collectively, brain oscillations dominated by specific frequency can be modulated by weak current, which sheds new light on the therapeutic advantages for older adults with co-occurring SCD and sleep disturbances. Particularly, high-definition so-tDCS applies a focal monophasic sinusoidal wave at a low frequency that can proficiently entrain brain

endogenous oscillations and modulate brain activities. Given the very limited therapeutic options for older adults suffering from SCD and sleep disturbances, we aim to conduct a randomized, double-blind, sham-controlled clinical trial to investigate the efficacy and sustainability of high-definition so-tDCS in the treatment of SCD and sleep disturbances and the clinical values of dynamic neurophysiological signals in predicting the treatment outcomes at individual level.

(ii) Research questions

1. Can we quantify the real-time neurophysiological signals during high-definition slow oscillatory transcranial direct current stimulation (so-tDCS) intervention?
2. Would repeated high-definition so-tDCS intervention alleviate subjective cognitive complaints and enhance sleep quality in older adults?
3. Would neurophysiological signals be associated with cognitive performance, sleep quality, and β-amyloid levels?
4. Can the features of neurophysiological signals (i.e., impedance) predict the treatment outcomes at individual level?

(iii) Research Methods

Research Plan

This study adopts the design of a randomized, double-blind, sham-controlled clinical trial of high-definition so-tDCS. Assessments will be conducted at baseline, 4th week, 8th week, and12^{th} week to evaluate the efficacy and sustainability of high-definition so-tDCS effects on subjective cognitive complaints, sleep quality, saliva Aβ and tau levels and domain-specific cognitive functions. Primary outcomes will be the changes in impedance, subjective cognitive complaints and sleep quality with so-tDCS intervention. Secondary outcomes will be the changes in cognitive functions and saliva β-amyloid levels, and the predictive relationships between the changes in impedance, subjective cognitive complaints, sleep quality and saliva Aβ and tau levels.

Methodology

Source of participants

Eligible participants will be recruited through existing local cohort [28], i.e., Hong Kong cohort of abnormal sleep in ageing population (HK-ASAP). The research team, including neuroscientist, psychiatrist, and geriatrician, will identify the eligible participants with subjective complaints of sleep and cognition, and determine their eligibility and availability to participate in this clinical trial study. Before joining in this study, both the participants and their families or caregivers will be briefed about the study and signed the written informed consent.

Eligible participants should meet the inclusion criteria as follows:

1. Chinese, right-handed, aged from 60 to 80 years.

2. Normal global cognition defined as a total score of the Montreal Cognitive Assessment Hong Kong version (HK MoCA) > 26 [17].

3. Subjective cognitive decline (SCD) is evaluated by a 10-item questionnaire that covers subjective cognitive complaints (SCC) in the core cognitive domains [31]. Participants will answer yes/no questions referring to the presence of cognitive deficits in the last six months.

The answers of SCC are codified as 0 (absence of complaints) or 1 (presence of complaints). Answers will be summed up and the total of complaints will be obtained ranging from 0 to 10. In this study, the status of SCD is defined as SCC total score > 1.

4. Sleep disturbances or poor sleep quality is assessed and defined with a total score of the Pittsburgh Sleep Quality Index (PSQI) greater than 5 [32].

5. No impairments in the activities of daily living.

Exclusion criteria include:

1. Previous diagnosis of age-related neurodegenerative diseases, such as Alzheimer's disease, Parkinson disease.
2. History of neurological diseases including brain tumor, and stroke.
3. History of psychiatric disorders including schizophrenia and depression.
4. Presence of positive neurological diseases, such as stroke, brain tumor, dementia.
5. Physically unable to attend the sessions of intervention.
6. Currently enrolled in another intervention study.
7. Currently taking a psychiatric or other medication that may affect sleep quality and cognition.

Pre-intervention data collection and quality control

Magnetic resonance imaging

High-resolution structural MRI (MRI) scans will be collected at the Prince of Wales Hospital using a 3.0 Tesla Siemens MAGNETOM Prisma MRI scanner for detecting the major neurological diseases, such as stroke, brain tumor. T1-weighted magnetization prepared rapid gradient echo (MPRAGE) sequence is applied to optimize the grey-white contrast. The MPRAGE sequence yields high quality isotropic images with the voxel size of 1 mm × 1 mm × 1 mm. The detailed parameters of this sequence are: axial acquisition with a 256×256×192 matrix, field of view (FOV)=230 mm, repetition time (TR)=2070 ms, echo time (TE)=3.93 ms, thickness=1 mm, no gap, flip angle=15°. All MRI scans will be imported to BrainSuite for quantitative analysis [35]. Surface-based morphometric measures, including cortical volume, thickness and folding, are employed as brain features to precisely localize the stimulation target, correlate with neurophysiological signals and control the baseline brain features in two randomized groups.

Intervention strategies

Apparatus and treatment setting

The so-tDCS is delivered by a battery driven direct current stimulator (DC-Stimulator Plus, NeuroConn, Ilmenau, Germany). The high-definition setting is applied by placing an anodal electrode over the center of the treatment target and placing four cathodal electrodes surrounded the anodal electrod. Each electrode in the "4 × 1" montages had a diameter of 1.2 cm. We place the anodal electrode over the center of left dorsolateral prefrontal cortex (DLPFC) (i.e., F3 according to the international 10-20 EEG system) as used in previous studies that investigated preserved cognition [36] and place the four return electrodes around anodal electrode. For safety and health considerations, single use sponge electrodes (1.2 cm) will be applied in this study. Participants will be instructed to relax during the intervention of high-definition so-tDCS.

Stimulation modalities

High-definition so-tDCS: 20 minutes at 0.75 Hz 2 milliamps [29,37].

Sham high-definition so-tDCS: With a same setting of high-definition as active so-tDCS, the stimulation of sham so-tDCS only lasts for 30 seconds. The electrodes will be left in place for a further 20 minutes. The purpose of this sham procedure is to mimic the transient skin sensation of tingling induced by current stimulation, but without producing any sustainable effects [29].

Intervention schedule

The duration of the intervention is four weeks, with three sessions per week, 20 minutes per session. All participants will receive a total of 12 sessions of high-definition so-tDCS.

Outcome assessments

Primary outcomes

1. Impedance during so-tDCS: a neurophysiological signal recorded and measured by a built-in wearable Galvanic Skin Response (GSR) sensor. The GSR sensor is placed on the fingers to detect the impedance of the skin when the participant is receiving the so-tDCS intervention (Appendix 4).
2. Subjective cognitive complaints (SCC): a 10-item questionnaire that covers subjective complaints in the core cognitive domains [31]. Participants will answer ten yes/no questions relating to the presence of cognitive complaints in the last six months. The answers of SCC are codified as 0 (no complaints) or 1 (presence of complaints). Answers will be summed up and the total of complaints will be obtained ranging from 0 to 10.
3. Subjective sleep quality: The 19-item self-reported questionnaire of the Pittsburgh Sleep Quality Index (PSQI) is employed to assess the subjective sleep quality. The 19 items produce seven component scores. The total score of these components yields a measure of subjective sleep quality or the status of sleep disturbances. The cutoff score of sleep disturbances is 5 or more [38].

Secondary outcomes

1. Actigraphic records were used to quantify sleep-wake cycle and estimate the objective sleep efficiency. The actigraph is about the size of a wristwatch and is usually worn on the wrist continuously for multiple days and nights, which can be used as an objective measurement of sleep quality and sleep-wake cycle in older adults.
2. Intra-individual variability of reaction time (IIV-RT), representing the within-person fluctuations, is calculated based on the performance of attention network test (ANT). For correcting the increased RT in elderly, Intraindividual coefficient of variation of reaction time (ICV-RT) is used for assessing the IIV-RT enumerated with the formula: ICV-RT = (Standard deviation of RT/mean RT) × 100 [17].
3. The levels of saliva Aβ: At the baseline and post-intervention time points, saliva samples will be collected at a consistent time of day to avoid circadian effects and will be kept on ice during the collection. The levels of Aβ42 and Aβ40 in the saliva samples will be quantified by enzyme-linked immunosorbent assay (ELISA)-type assays (Aurin Biotech, Inc.) [39]. Each sample will be analyzed in duplicate.

Assessment schedule

The comprehensive assessments, including subjective cognitive complaints, sleep quality, cognitive function, adverse events and saliva Aβ42 and Aβ40, will be conducted at one week before intervention (baseline, T0), 4th week (T1), 8th week (T2), and 12th week (T3).

Sample Size Estimation

The sample size is estimated using G*POWER. Focusing on our study objectives, the estimation will be evaluated by comparing the PSQI total score across randomized groups and time. The potential effect size of improved subjective sleep quality induced by repeated active tDCS is estimated from our previous findings of a tDCS intervention conducted in mild neurocognitive disorder due to Alzheimer's disease [28]. A 4-week course tDCS showed significant positive effects on the subjective sleep quality (Score change of PSQI total score: 2.36±1.51). Based on the proposed statistical analysis of the fixed effects of so-tDCS on the PSQI total score, we set the desired power (1-β) as 0.8 and confidence level as 0.95. Thirty-six participants in each intervention group will be needed to achieve a statistical power of 0.8. Considering the dropout rate of around 13% in clinical trial [28], 40 participants in each group, a total of 80 participants, should be recruited in this clinical trial.

Statistical Analyses

Analyses will be on an intention-to-treat basis. Linear mixed models will be applied to assess and quantify the differences between the pre- and post-intervention time points on the primary and secondary outcome measures. This statistical method will facilitate the inclusion of participants with missing data. Time (i.e., follow-up period) will be modelled as fixed effects. Participants will be modelled as random effects at different time points. Score changes of subjective cognitive complaints, neurophysiological features, sleep quality, cognition and saliva Aβ and tau levels from baseline to follow-up time points will be tested with occasions (i.e., time points) at level one and participants at level two. For better controlling the practice effects, generalized estimating equations will be used in the analysis of repeated measurements of treatment outcomes. Covariates identified from baseline differences will be entered in the regression model. Secondary analyses of the predictive relationships between cognitive complaints, sleep quality, β-amyloid levels and neurophysiological features will be performed. Statistical significance will be set at 2-sided $p < 0.05$. Computations will be performed using R Studio (version 1.1.456).

Ethical consideration

Ethics standards will be strictly followed by providing informed consent and respecting anonymity, privacy and confidentiality. Participants will be recruited if the participants are considered mentally fit to sign consent. No personal identity, including name, birth date, mobile numbers, will be revealed in any reports or publications. Participants can withdraw anytime without interference from any future service use. For those who have any medical concerns during this clinical trial, they will be advised to seek help from clinicians.

Ethics approval from the Clinical Research Ethics Committee (The Joint CUHK-NTEC CREC) will be obtained before the commencement of this study. The protocol will also be registered with the CCT Clinical Trials Registry of the Chinese University of Hong Kong and linked to the ChiCTR, WHO-ICTRP China Primary Registry. The reporting of trial will follow the requirements of major international academic journals. The study will comply with the Declaration of Helsinki and the Good Clinical Practice guidelines of the International Conference on Harmonisation of technical requirements for registration of pharmaceuticals for human use (ICH-GCP).

References

[1] Jessen, F., Amariglio, R. E., Buckley, R. F., van der Flier, W. M., Han, Y., Molinuevo, J. L., & Wagner, M. (2020). The characterisation of subjective cognitive decline. The Lancet Neurology, 19(3), 271-278.

[2] Rabin, L. A., Smart, C. M., & Amariglio, R. E. (2017). Subjective cognitive decline in preclinical Alzheimer's disease. Annual review of clinical psychology, 13, 369-396.

[3] Jonker, C., Geerlings, M. I., & Schmand, B. (2000). Are memory complaints predictive for dementia? A review of clinical and population-based studies. International journal of geriatric psychiatry, 15(11), 983-991.

[4] Reisberg, B., & Gauthier, S. (2008). Current evidence for subjective cognitive impairment (SCI) as the pre-mild cognitive impairment (MCI) stage of subsequently manifest Alzheimer's disease. International psychogeriatrics, 20(1), 1-16.

[5] Stewart, R. (2012). Subjective cognitive impairment. Current opinion in psychiatry, 25(6), 445-450.

[6] Liew, T. M. (2020). Subjective cognitive decline, anxiety symptoms, and the risk of mild cognitive impairment and dementia. Alzheimer's research & therapy, 12(1), 1-9.

[7] Liew, T. M. (2019). Depression, subjective cognitive decline, and the risk of neurocognitive disorders. Alzheimer's research & therapy, 11(1), 1-8.

[8] Exalto, L. G., Hendriksen, H. M., Barkhof, F., van den Bosch, K. A., Ebenau, J. L., van Leeuwenstijn-Koopman, M., & van der Flier, W. M. (2022). Subjective cognitive decline and self-reported sleep problems: The SCIENCe project. Alzheimer's & Dementia: Diagnosis, Assessment & Disease Monitoring, 14(1), e12287.

[9] Tsapanou, A., Vlachos, G. S., Cosentino, S., Gu, Y., Manly, J. J., Brickman, A. M., & Mayeux, R. (2019). Sleep and subjective cognitive decline in cognitively healthy elderly: results from two cohorts. Journal of sleep research, 28(5), e12759.

[10] Wen, C., Hu, H., Ou, Y. N., Bi, Y. L., Ma, Y. H., Tan, L., & Yu, J. T. (2021). Risk factors for subjective cognitive decline: the CABLE study. Translational Psychiatry, 11(1), 576.

[11] Shi, L., Chen, S. J., Ma, M. Y., Bao, Y. P., Han, Y., Wang, Y. M., & Lu, L. (2018). Sleep disturbances increase the risk of dementia: a systematic review and meta-analysis. Sleep medicine reviews, 40, 4-16.

[12] La Joie, R., Perrotin, A., Egret, S., Pasquier, F., Tomadesso, C., Mézenge, F., & Chételat, G. (2016). Qualitative and quantitative assessment of self-reported cognitive difficulties in nondemented elders: association with medical help seeking, cognitive deficits, and β-amyloid imaging. Alzheimer's & Dementia: Diagnosis, Assessment & Disease Monitoring, 5, 23-34.

[13] Ismail, Z., McGirr, A., Gill, S., Hu, S., Forkert, N. D., & Smith, E. E. (2021). Mild behavioral impairment and subjective cognitive decline predict cognitive and functional decline. Journal of Alzheimer's Disease, 80(1), 459.

[14] Perrotin, A., Mormino, E. C., Madison, C. M., Hayenga, A. O., & Jagust, W. J. (2012). Subjective cognition and amyloid deposition imaging: a Pittsburgh Compound B positron emission tomography study in normal elderly individuals. Archives of neurology, 69(2), 223-229.

[15] Vogel, J. W., Doležalová, M. V., La Joie, R., Marks, S. M., Schwimmer, H. D., Landau, S. M., & Jagust, W. J. (2017). Subjective cognitive decline and β-amyloid burden predict cognitive change in healthy elderly. Neurology, 89(19), 2002-2009.
[16] Bielak, A. A., Cherbuin, N., Bunce, D., & Anstey, K. J. (2014). Intraindividual variability is a fundamental phenomenon of aging: Evidence from an 8-year longitudinal study across young, middle, and older adulthood. Developmental psychology, 50(1), 143.
[17] Lu, H., Fung, A. W., Chan, S. S., & Lam, L. C. (2016). Disturbance of attention network functions in Chinese healthy older adults: an intra-individual perspective. International psychogeriatrics, 28(2), 291-301.
[18] Ngo, H. V. V., Claassen, J., & Dresler, M. (2020). Sleep: slow wave activity predicts amyloid-β accumulation. Current Biology, 30(22), R1371-R1373.
[19] Kylkilahti, T. M., Berends, E., Ramos, M., Shanbhag, N. C., Töger, J., Markenroth Bloch, K., & Lundgaard, I. (2021). Achieving brain clearance and preventing neurodegenerative diseases—A glymphatic perspective. Journal of Cerebral Blood Flow & Metabolism, 41(9), 2137-2149.
[20] Etter, G., van der Veldt, S., Manseau, F., Zarrinkoub, I., Trillaud-Doppia, E., & Williams, S. (2019). Optogenetic gamma stimulation rescues memory impairments in an Alzheimer's disease mouse model. Nature communications, 10(1), 1-11.
[21] Manousakis, J. E., Nicholas, C., Scovelle, A. J., Naismith, S. L., & Anderson, C. (2019). Associations between sleep and verbal memory in subjective cognitive decline: A role for semantic clustering. Neurobiology of learning and memory, 166, 107086.
[22] Winer, J. R., Mander, B. A., Kumar, S., Reed, M., Baker, S. L., Jagust, W. J., & Walker, M. P. (2020). Sleep disturbance forecasts β-amyloid accumulation across subsequent years. Current Biology, 30(21), 4291-4298.
[23] Kim, S. Y., Pardilla-Delgado, E., & Alger, S. E. (2017). Enhancing memory consolidation through slow oscillation and spindle synchronization. Journal of Neuroscience, 37(48), 11517-11519.
[24] Ketz, N., Jones, A. P., Bryant, N. B., Clark, V. P., & Pilly, P. K. (2018). Closed-loop slow-wave tACS improves sleep-dependent long-term memory generalization by modulating endogenous oscillations. Journal of Neuroscience, 38(33), 7314-7326.
[25] Yao, S., Liu, Y., Zheng, X., Zhang, Y., Cui, S., Tang, C., & Xu, N. (2020). Do nonpharmacological interventions prevent cognitive decline? a systematic review and meta-analysis. Translational psychiatry, 10(1), 1-11.
[26] Smart, C. M., Karr, J. E., Areshenkoff, C. N., Rabin, L. A., Hudon, C., Gates, N., & Subjective Cognitive Decline Initiative. (2017). Non-pharmacologic interventions for older adults with subjective cognitive decline: systematic review, meta-analysis, and preliminary recommendations. Neuropsychology review, 27(3), 245-257.
[27] Summers, J. J., Kang, N., & Cauraugh, J. H. (2016). Does transcranial direct current stimulation enhance cognitive and motor functions in the ageing brain? A systematic review and meta-analysis. Ageing research reviews, 25, 42-54.
[28] Lu, H., Chan, S. S. M., Chan, W. C., Lin, C., Cheng, C. P. W., & Linda Chiu Wa, L. (2019). Randomized controlled trial of tDCS on cognition in 201 seniors with mild neurocognitive disorder. Annals of clinical and translational neurology, 6(10), 1938-1948

[29] Lu, H., & Lam, L. C. W. (2019). Cathodal skin lesions induced by transcranial direct current stimulation (tDCS). Neuromodulation: Technology at the Neural Interface, 22(8), 989-991.
[30] Lam, L. C. W., Wong, C. S. M., Wang, M. J., Chan, W. C., Chen, E. Y. H., Ng, R. M. K., & Bebbington, P. (2015). Prevalence, psychosocial correlates and service utilization of depressive and anxiety disorders in Hong Kong: the Hong Kong Mental Morbidity Survey (HKMMS). Social psychiatry and psychiatric epidemiology, 50(9), 1379-1388.
[31] Markova, H., Andel, R., Stepankova, H., Kopecek, M., Nikolai, T., Hort, J., & Vyhnalek, M. (2017). Subjective cognitive complaints in cognitively healthy older adults and their relationship to cognitive performance and depressive symptoms. Journal of Alzheimer's Disease, 59(3), 871-881.
[32] Lu, H., Li, J., Yang, N. S., Lam, L. C. W., Ma, S. L., Wing, Y. K., & Zhang, L. (2023). Using gamma-band transcranial alternating current stimulation (tACS) to improve sleep quality and cognition in patients with mild neurocognitive disorders due to Alzheimer’s disease: A study protocol for a randomized controlled trial. PLoS One, 18(8), e0289591.
[33] Lu, H., Ma, S. L., Chan, S. S. M., & Lam, L. C. W. (2016). The effects of apolipoprotein ε 4 on aging brain in cognitively normal Chinese elderly: a surface-based morphometry study. International Psychogeriatrics, 28(9), 1503-1511.
[34] Faghy, M. A., Whitsel, L., Arena, R., Smith, A., & Ashton, R. E. (2023). A united approach to promoting healthy living behaviours and associated health outcomes: a global call for policymakers and decision makers. Journal of Public Health Policy, 44(2), 285.
[35] Lu, H., Chan, S. S. M., Ma, S., Lin, C., Mok, V. C. T., Shi, L., & Lam, L. C. W. (2022). Clinical and radiomic features for predicting the treatment response of repetitive transcranial magnetic stimulation in major neurocognitive disorder: Results from a randomized controlled trial. Human Brain Mapping, 43(18), 5579-5592.
[36] André, S., Heinrich, S., Kayser, F., Menzler, K., Kesselring, J., Khader, P. H., & Mylius, V. (2016). At-home tDCS of the left dorsolateral prefrontal cortex improves visual short-term memory in mild vascular dementia. Journal of the neurological sciences, 369, 185-190.
[37] Bueno-Lopez, A., Eggert, T., Dorn, H., & Danker-Hopfe, H. (2019). Slow oscillatory transcranial direct current stimulation (so-tDCS) during slow wave sleep has no effects on declarative memory in healthy young subjects. Brain stimulation, 12(4), 948-958.
[38] Chen, H. C., Hsu, N. W., & Chou, P. (2020). Subgrouping poor sleep quality in community-dwelling older adults with latent class analysis-the Yilan Study, Taiwan. Scientific reports, 10(1), 5432.
[39] Verberk, I. M., Hendriksen, H. M., van Harten, A. C., Wesselman, L. M., Verfaillie, S. C., van den Bosch, K. A., Philip Scheltens, Charlotte E Teunissen & Van der Flier, W. M. (2020). Saliva amyloid is associated with the rate of cognitive decline in cognitively normal elderly: the SCIENCe project. Neurobiology of Aging, 89, 99-107.
